# TH1 Cell Frequency and Neutrophil-to-Lymphocyte Ratio in Hemodialysis: Potential Contributions to Patient Monitoring

**DOI:** 10.3390/biomedicines12102188

**Published:** 2024-09-26

**Authors:** Inês Rodrigues Barreto, Andreia Monteiro, Ernesto Fernandes Rocha, Catarina Reis Santos, Ana Mafalda Fonseca

**Affiliations:** 1 CICS-UBI—Health Sciences Research Centre, University of Beira Interior, Av. Infante D. Henrique, 6200-506 Covilhã, Portugal; ines.r.barreto@ubi.pt; 2 Faculty of Health Sciences, University of Beira Interior, 6200-506 Covilhã, Portugal; asmonteiro@fcsaude.ubi.pt; 3 Clinical Pathology Service, Centro Hospitalar Universitário da Cova da Beira (CHUCB), Alameda Pêro da Covilhã, 6200-251 Covilhã, Portugal; 4 ULS Castelo Branco, Avenida Pedro Alvares Cabral, 6000-085 Castelo Branco, Portugal; erocha55@gmail.com

**Keywords:** chronic renal disease, hemodialysis, Th1 cells, NLR

## Abstract

**Introduction:** Patients undergoing hemodialysis (HD) often exhibit an impaired cellular immune response, which may contribute to an increased susceptibility to infections and other complications. Th1 cells, a subset of T-helper cells, play a crucial role in cellular immunity. However, the modulation of Th1 cells by HD treatment remains unclear. **Objective:** This study aims to investigate the levels of circulating T cells, especially Th1 cells, and the neutrophil-to-lymphocyte ratio (NLR) in HD patients. **Methods**: We recruited 26 HD patients and 10 healthy volunteers. Demographical data were collected, and peripheral blood samples were analyzed. Absolute blood cell counts were determined, and T-cell populations were identified using flow cytometry. Th1 cells were defined as IFN-γ-producing CD4^+^ T cells after in vitro activation, and NLR was calculated through the ratio between the neutrophil and lymphocyte counts measured in peripheral blood. **Results:** We have observed a significant decrease in Th1 subpopulation frequency in HD patients, as well as significant correlations between immunological and demographic parameters, among which are the NLR values and the absolute values of T-cell subsets. **Conclusions:** These results seem to clarify the role of Th1 cells in modulating the immune responses of hemodialysis-treated patients, potentially considering its frequency as an indicator for CKD development.

## 1. Introduction

Chronic kidney disease (CKD) is a complex heterogeneous disease, with increasing prevalence [1], characterized by the progressive loss of kidney function, ultimately leading to kidney failure [2,3]. Both physiological and lifestyle-related risk factors seem to adversely impact the development of the disease [4], and, even though the etiology is diverse, diabetes and hypertension constitute the most common primary causes of CKD [5], independently accounting for a higher incidence of adverse cardiovascular events [6]. 

Over time, patients silently progress towards a state of kidney failure, in which they will need to be submitted to either a renal transplant or kidney replacement therapy, like hemodialysis (HD) [7]. To restore the electrolyte balance and remove the metabolic waste products from blood, HD relies on the principles of solute diffusion across a synthetic semipermeable membrane, through an extracorporeal circuit [8].

Although HD itself can be seen as the root cause of many of the complications associated with these patients, the renal damage appears to be, in part, modulated by a persistent low-grade inflammation and impaired innate and adaptative immune responses [9,10]. Upon antigen interaction, naïve CD4^+^ T cells can differentiate into distinct T-helper (Th) effector subpopulations, based on the expression of a specific cytokine profile. Th1 cells are responsible for the response against intracellular pathogens, through the secretion of IFN-γ and IL-12, but can also be regarded as an inductor of some autoimmune diseases [11,12].

Indeed, T cells seem to be responsible for the immune dysfunction CKD patients are exposed to, many of them showing lower percentages of CD4^+^ and CD8^+^ T cells, leading to an inverted cell ratio, as well as an altered Th1/Th2 balance, with contradictory results [13,14]. Besides that, an accumulation of terminally differentiated T cells is regularly observed, with a marked reduction in naïve T cells [15,16]. The deterioration of kidney function also seems to affect the levels of circulating cytokines, significantly increasing the pro-inflammatory ones such as IL-1β, TNF-α, and IL-6, and reducing anti-inflammatory IL-10 [17,18,19]. Nevertheless, it should be noted that the HD procedure itself, due to the prolonged exposure to artificial components, also compromises and further aggravates renal damage [20]. 

In the last few years, complementary biomarkers to measure systemic inflammation in CKD patients have been sought. Reflecting the balance between innate and adaptative immunity, the neutrophil-to-lymphocyte ratio (NLR) has been proposed as a prognostic tool [21], being associated with poor outcomes in cardiovascular diseases [22], infections [23], and cancer [24]. This tendency seems to be no exception in CKD, as some have reported this ratio to be valuable in predicting the disease’s progression and management [25,26,27]. To note, it does not seem obvious or correct to identify a single, overall NLR value associated with a poor prognosis in studies on CDK patients because each study focuses on different groups (e.g., dialysis patients vs. non-dialysis patients). The results vary significantly between these groups, and a single value could obscure these differences. Yet, NLR values higher than 2.42 were reported to be linked with increased inflammation [25,26] and more severe CDK, namely, as patients approach needing dialysis, increasing NLR values in the range 1.35–4.29) [27]. However, while previous research underscores the importance of NLR measurement in monitoring HD patients, studies that simultaneously analyze both NLR and Th1 cells (a major role in inflammatory adaptive responses) in the same individuals are lacking. Importantly, the NLR value has low cost and it is easy to obtain from a complete blood count. 

The main goal of this work was to further contribute to the understanding of how the frequency of the peripheral Th1 subpopulation changes in CKD patients submitted to HD, comparing it with a control group. We then investigated how cell subsets could be influenced by dialysis and demographic-related characteristics.

## 2. Materials and Methods

### 2.1. Patients and Healthy Controls

Twenty-six HD patients were recruited from the Nephrology Service of ULS Castelo Branco, EPE-Hospital Amato Lusitano. The inclusion criteria required participants to have been undergoing HD treatment for at least three months and to have a confirmed diagnosis of controlled hypertension (level 1: systolic reading less than 139 mm Hg and diastolic reading less than 89 mm Hg). We excluded patients who were treated with immunosuppressors or who were suffering from any infectious or inflammatory process, whether acute or chronic. Dialysis technique was performed via either an arteriovenous fistula or a central dialysis catheter, three times a week, with a four-hour duration for each session, and using a polysulfone high-flux dialyzer (FX CorDiax 600 or FX CorDiax 800, Fresenius Medical Care, Bad Homburg, Germany). The control group was constituted by ten healthy sex-matched volunteers with no medical history of hypertension, diabetes, or immune-related disorders. Potential ongoing inflammatory process were also ruled out, according to the measurement of serum C-reactive protein (CRP) levels.

Prior to the mid-week HD session, peripheral blood (PB) samples were drawn into heparin tubes for subsequent flow cytometry analysis. To determine absolute blood cell counts, Yumizen H2500 (Horiba, Kyoto, Japan) hematological cell analyzer was used.

The Ethics Committee of ULS Castelo Branco, EPE-Hospital Amato Lusitano (Castelo Branco, Portugal) approved the study and written informed consent was provided to both HD patients and volunteers.

### 2.2. Cell Activation

PB samples were, firstly, activated with a Cell Activation Cocktail (with Brefeldin A) (BioLegend, San Diego, CA, USA), containing an optimized concentration of phorbol 12-myristate-13-acetate (PMA), ionomycin, and Brefeldin A. A total of 500 μL of each PB sample was diluted in 500 μL of RPMI-1640 medium (Gibco, Paisley, Scotland, UK), and 1 μL of the referred cocktail was added, followed by an 4 h incubation period at 37 °C in a sterile environment with a 5% CO_2_ humid atmosphere.

### 2.3. Peripheral Blood T-Cell Subset Phenotyping

After the activation period, the PB samples were aliquoted in two tubes, one identified as control (without antibodies). For staining intracellular cytokines, Cytofix/Cytoperm^TM^ Fixation/Permeabilization Kit (BD Biosciences, San Jose, CA, USA), a permeabilization and fixation protocol was followed. According to the manufacturer’s instructions, samples were initially stained for the surface antigens with the following mouse anti-human monoclonal antibodies: anti-CD3-APC (Allophycocyanin, clone OKT3, isotype IgG2a, κ; BioLegend, San Diego, CA, USA) and anti-CD4-PerCP (Peridinin chlorophyll-A protein/Cyanine5.5, clone RPA-T4, isotype IgG1, κ; BioLegend, San Diego, CA, USA). The staining was carried out in the dark at room temperature (RT) for 15 min, after which samples were washed with 2 mL of PBS and centrifuged at 2000 rpm for 5 min. For the next step, cell pellet was resuspended in 500 μL of Fixation/Permeabilization solution, and, following previous conditions, incubated for 20 min and then centrifugated at 2000 rpm for 5 min. As part of a washing step, 2 mL of BD Perm/Wash^TM^ buffer (diluted 1:10 in distilled H_2_O) were added, followed by a 10 min incubation step in the dark at RT and a centrifugation at 2000 rpm for 5 min. Intracellular staining was performed next with 5 μL of anti-IFN-γ-FITC (Fluorescein isothiocyanate, clone 4S.B3, isotype IgG1, κ; BioLegend, San Diego, CA, USA) being added to the cell pellet and incubated for 30 min in the previous conditions. After another washing step, cell pellet was resuspended in less than 500 μL of PBS and samples were immediately acquired in a FACSCalibur^TM^ (BD Biosciences, San Jose, CA, USA) flow cytometer. For each sample and whenever possible, 1 × 10^4^ events were acquired and analyzed using Infinicyt^TM^ software (version 1.8, Cytognos SL, Salamanca, Spain).

**Figure 1:**
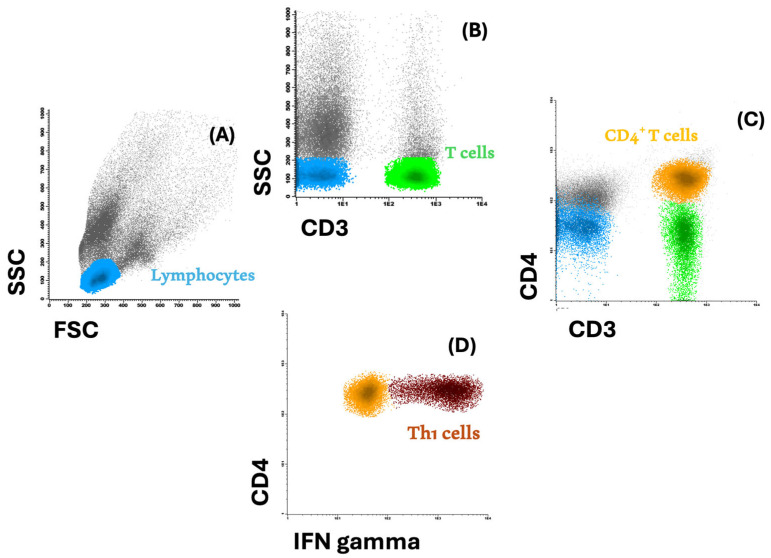
Flow cytometry analysis strategy. (**A**) The total lymphocyte population (in blue) was identified based on forward (FSC) and side (SSC) scatter properties; (**B**) within the total lymphocytes’ population based on the expression of CD3, T cells can be identified (in green), and, (**C**) subsequently, CD4^+^ (in orange), and (**D**) Th1 cells were then identified in the CD4^+^ T-cell compartment, based on the expression of IFN-γ (in brown).

Total lymphocyte population was firstly identified based on forward (FSC) and side (SSC) scatter properties. Within this cell population, the identification of T cells and, subsequently, of CD4^+^ T cells was based on the expression of CD3 and CD4, respectively. CD8^+^-enriched T cells were identified by exclusion as CD4^−^ CD3^+^ T cells. It is important to be aware that other T-cell populations, although of minor representativeness, such as γδ T cells, may also lack CD4 expression. Given the expression of IFN-γ, Th1 cells were identified as CD4^+^ IFN-γ^+^ [28]. Figure 1 exemplifies the employed strategy of flow cytometry analysis to identify T-cell subpopulations.

### 2.4. Statistical Analysis

Statistical analysis was performed using SPSS software (version 28.0.1.0, IBM, Armonk, NY, USA). Normality was tested by using Shapiro–Wilk test. Data are expressed as mean ± standard deviation (SD). To determine the significance of the differences between PB samples of HD patients and controls, independent-sample *t*-test was used. Pearson rank correlation test was performed to determine the associations between immunological and clinical characteristics of HD patients. A *p*-value of *p* < 0.05 was considered statistically significant.

## 3. Results

### 3.1. General Characteristics of the Subjects

Table 1 summarizes the demographic and clinical characteristics of HD patients and controls included in this study. Age, gender, blood leukocyte counts, blood neutrophil counts, CRP, and serum creatinine were the available data concerning the control group. 

The male gender is the most represented among both groups, encompassing more than 60% of the studied population. Besides this, the data also show that HD patients had a significantly higher mean age than the control group (*p* < 0.001). The same tendency was also observed regarding serum creatinine and CPR values in HD patients, in comparison with the control group (*p* < 0.001 and *p* = 0.012, respectively). On the contrary, blood neutrophil counts are significantly diminished in HD patients, when compared with the control group (*p* < 0.001). The same tendency is observed regarding NLR values in HD patients (*p* = 0.013).

Focusing on HD patients, it is relevant to note that the development of pyelonephritis is due to acquired obstructive uropathy, while the diagnosis of renal vascular disease is due to hypertension, without a primary renal disease associated. The “Other etiology” category includes diseases such as multisystemic disease, chronic renal failure of uncertain etiology, pyelonephritis due to urolithiasis, and polycystic kidneys, among others. Dialysis adequacy was evaluated by the measurement of urea clearance using the equation Kt/V, where K = urea clearance, t = time on dialysis, and V = volume of distribution. According to KDIGO guidelines, it should be 1.4 per hemodialysis session for patients treated thrice weekly [29]. 

Comorbidities are also significant for the progression of CKD and may negatively impact the disease. Since this information is not available for all patients, we decided not to include it. Nevertheless, of note, some patients of the studied HD cohort were, for example, diagnosed with diabetes, congestive heart failure, cerebrovascular diseases, or peripheral vascular diseases. A similar explanation can be applied regarding the medication prescribed to these patients, as it intends to treat their specific conditions. Besides that, all HD patients are treated with ferric oxide and vitamin D. 

### 3.2. T-Cell Subsets in HD Patients and Controls

When we compare T-cell subsets in HD patients and controls, we find that HD patients presented a greater total lymphocyte count. Conversely, both Th1-cell frequency and absolute values are significantly reduced among HD patients, in comparison with the control group (*p* < 0.001 and *p* = 0.012, respectively) (Table 2).

### 3.3. Correlations between Immunological and Clinical Characteristics of HD Patients

**Figure 2:**
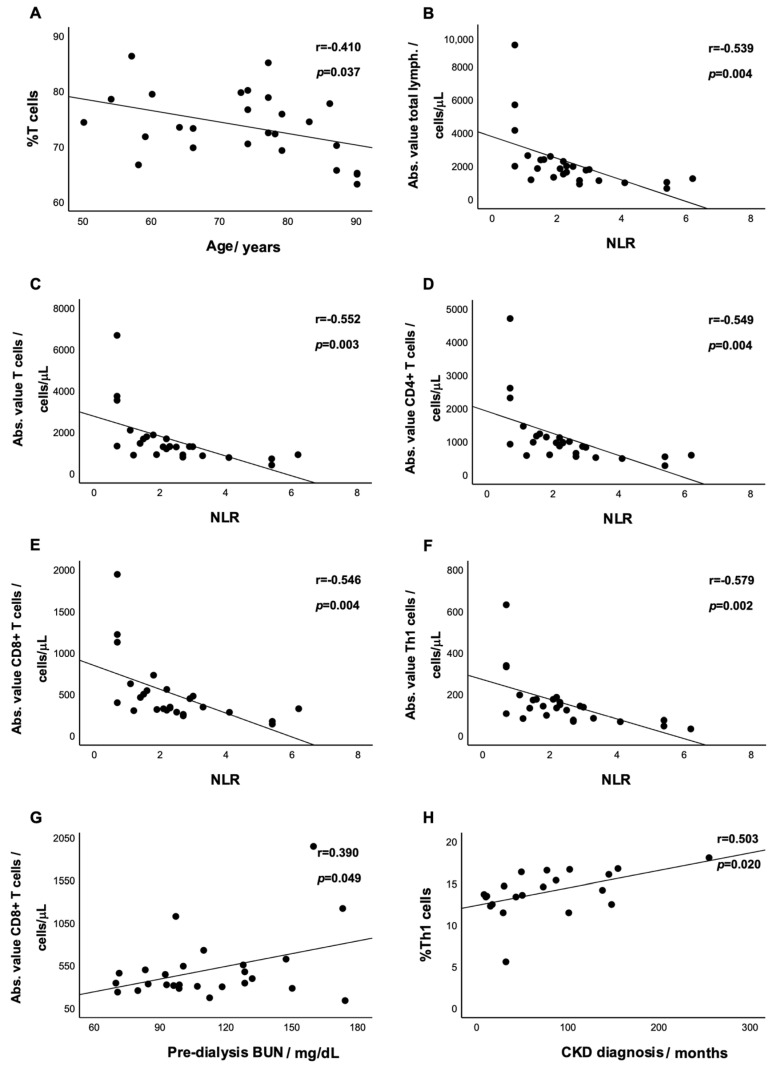
Scatter plots for the correlations between HD patients’ age and T-cell frequency (**A**), NLR value and total lymphocytes absolute value (**B**), NLR value and T-cell absolute value (**C**), NLR value and CD4^+^ T-cell absolute value (**D**), NLR value and CD8^+^-enriched T-cell absolute value (**E**), NLR value and Th1-cell absolute value (**F**), pre-dialysis BUN value and CD8^+^-enriched T-cell absolute value (**G**), and CDK diagnosis and Th1-cell frequency (**H**).

Based on the results obtained so far, we decided to further analyze the relationships between age, sex, CKD diagnosis, HD vintage, Kt/V, NLR, CRP, serum creatinine, pre- and post-dialysis BUN, and the frequencies and absolute values of T-cell subsets. The HD patients’ ages were negatively correlated with the frequency of T cells (r = −0.410, *p* = 0.037, Figure 2A). The same pattern was observed regarding NLR values, which were negatively correlated with total lymphocytes, T cells, CD4^+^ and CD8^+^ T, and Th1 cell absolute values (r = −0.539, *p* = 0.004, Figure 2B; r = −0.552, *p* = 0.003, Figure 2C; r = −0.549, *p* = 0.004, Figure 2D; r = −0.546, *p* = 0.004, Figure 2E; and r = −0.579, *p* = 0.002, Figure 2F, respectively). CD8^+^ T-cell absolute values were also positively correlated with pre-dialysis BUN (r = 0.390, *p* = 0.049, Figure 2G). Likewise, the CDK diagnosis period was positively correlated with the frequency of Th1 cells (r = 0.503, *p* = 0.020, Figure 2H).

## 4. Discussion

There seems to be a consensus in the literature regarding the changes observed in the immune system of hemodialysis-treated CKD patients. Nevertheless, the contribution of Th1 cells to the immune response in these patients is not completely known.

In the present study, we demonstrated that the total lymphocyte counts were decreased in HD patients, when compared with a control group. However, the main finding of this work falls on the frequency of Th1 cells, significantly decreased in HD patients. As previously mentioned, conflicting results have been published regarding this subpopulation. While some authors have shown a significantly increased frequency of CD4^+^ T cells in HD patients, characterized by a Th1-type cytokine secretion pattern [14,30], others report that this frequency was comparable between HD patients and controls [13,31,32]. These differences in the literature probably reflect the specific clinical characteristics of the populations analyzed in the different studies, including the duration of HD treatment (months/years). Importantly, since Th1 cells play a crucial role in the immune response by producing cytokines that activate macrophages and promote the clearance of intracellular pathogens [33], a reduction in their population can contribute to the increased susceptibility to infections. We should not exclude that the reduction observed in Th1 numbers could be due to the dialysis membrane [34]. Addressing this subject in future longitudinal studies is important to better understand if indeed there is a negative correlation between Th1 cell numbers and infections, and the mechanisms underlying this susceptibility, in order to develop targeted interventions. Moreover, the further understanding of how different vascular access types might influence Th1 cell levels will also clarify the potential contribution of studying Th1 cells in HD patients. 

Nevertheless, it is important to note that, while IFN-γ levels may be significantly increased in HD patients, its production is not entirely attributed to Th1 cells, meaning other cell subpopulations could be responsible for the pro-inflammatory environment [31,35]. These results clearly show that a Th1/Th2 imbalance is present among HD patients, but further clarification is needed, since our study did not evaluate the frequency of Th2 nor any related cytokine. The same seems to apply to Th17/Treg ratio, whose imbalance may contribute to the development of inflammation [36,37]. 

Regarding the reduced number of neutrophil counts in HD patients when compared with healthy controls, suggesting an impairment in innate immune system, Zhang and colleagues obtained similar results where patients showed an observable reduction in neutrophils in ESRD patients, although it was not significantly different [36,37]. One should consider that the renal dysfunction present in these patients could be impacting the hemopoiesis [38], or the neutrophil aging process in the circulation and migration into inflamed tissues [33]. Moreover, it has been documented that HD membranes may induce the formation of platelet–neutrophil complexes [39], or the acceleration of neutrophil apoptosis [40]. 

It is important to note that the participants in the control group were not age-matched with the HD patients. Therefore, we must consider the effects of immunosenescence, which is the gradual deterioration of both the innate and adaptive immune systems associated with aging. The hallmarks of immunosenescence include a decline in T-cell production associated with age due to thymic degeneration, abnormal T-cell metabolism, changes in the proportion of T subpopulations, and a senescence-associated secretory phenotype (SASP)-mediated chronic low-grade inflammatory environment [41]. Regarding neutrophils, it has been shown that their absolute numbers do not change significantly in immunosenescence between adults and the elderly [42]. In what concerns lymphocyte counts, particularly the CD4+ T-cell population, existing evidence indicates a reduction with age [42,43,44,45], but, most probably, these changes are not significant enough to justify a modification of our results. Consequently, we could expect that the NLR in elderly healthy subjects would be even higher than the one we found [44,46]. 

To further explore and possibly explain what could contribute to the immune dysregulation, we decided to analyze the relationship between immunological and clinical characteristics among HD patients. It should be noted that correlations tell us how strong the association between the variables is, but not the cause and effect in that relationship. As expected, HD patients’ ages were negatively correlated with the frequency of T cells [47,48]. The deterioration of immune responses, especially the T-cell compartment, is expected with healthy aging, making the elderly population more susceptible to inflammatory diseases [49]. In the context of CKD, several studies have demonstrated that these patients are subjected to premature immunological changes, resembling those of normal senescence [15,50]. This premature aging is characterized by a significant T-cell receptor (TCR) diversity reduction, repertoire skew, and an accumulation of the CD4^+^ CD28^−^ subset, and is associated with cardiovascular diseases, accounting as a predictor of poor survival among HD patients, directly implicating disease progression [51,52,53]. 

We addressed the correlation between NLR and T-cell populations in HD patients and found NLR values were negatively correlated with total lymphocytes, T cells, CD4+ and CD8+ T, and Th1-cell absolute values. There is already evidence regarding its application as a valuable tool to assess the occurrence of complications in HD patients [54]. However, the literature is still sparse in analyzing such a relationship between the systemic inflammatory marker and T-cell subpopulations. Nevertheless, these results seem to be in line with previously published works that associate higher NLR values with higher inflammatory cytokines levels and with advanced CKD stages, indicating it can be considered a potential biomarker for predicting inflammation in CKD, as well as its progression [25,26,27]. Likewise, besides an association with nutritional values, low NLR values also seem to be related with lower hospitalization rates in HD patients with diabetes [55].

In addition, although pronounced, we observed a positive relationship between pre-dialysis BUN values and the absolute values of CD8^+^-enriched T cells. Traditionally associated with changes in renal perfusion, this biomarker has been looked at as a prognostic tool for heart failure [56], as well as a means to evaluate kidney function and hemodialysis efficacy, since it is inversely related with the glomerular filtration rate [57]. Interestingly, some have also shown a positive correlation between this marker and the nutritional status of CDK patients on hemodialysis, indicating pre-dialysis BUN values have the potential to improve malnutrition in HD patients [58]. We could argue we have observed a similar tendency in our study, since previous studies have shown that not only do dialysis adequacy markers relate to nutritional status in HD [59], immune functions are also modulated and influenced by nutrition parameters [60]. Due to its relevance, further clarification is needed regarding this subject to determine its clinical importance to disease development.

Lastly, we have found that Th1 cells’ frequency positively correlated with the CKD diagnosis period. As previously stated, immune cells indeed influence disease progression, and its impairment can accelerate CKD development towards complete kidney failure. Iio et al., has concluded that increased highly differentiated CD4^+^ T cells could predict renal outcomes, meaning that monitoring this subpopulation might help slow down the expected CKD progression [61]. Although it is necessary to conduct more studies, it seems plausible to consider the Th1 frequency as a predictor of CKD worsening, as well as other clinical outcomes.

Overall, while our study seems to show some promising results and to contribute to the clarification of the role of Th1 cells in CDK patients on hemodialysis, we are aware there were some limitations, including the number of individuals studied in the disease group, which directly impacts the wide-ranging expression levels observed for this population; and the fact that the control group was not age-matched. Furthermore, the fact that we decided to study CKD patients as a whole and not subdivide them according to the disease primary cause may also have influenced final results, since specific factors contribute differently to each disease. In the future, further attention should be given to these, as well as to their relationship with immunological parameters. Given that peripheral T cells encompass a heterogeneous group of cell types, including naive, effector, and memory cells, a comprehensive study of these T-cell compartments would be relevant in this context. Moreover, the measurement of inflammaging-related and regulatory serum cytokines can contribute to a deeper study of this in patients as well as longitudinal studies. Additionally, functional studies not only with T cells but also with neutrophils are needed to investigate the mechanistic pathways underlying these findings.

We believe that understanding and utilizing the NLR and peripheral Th1 frequency in clinical practice can provide a clearer picture of the inflammatory status of CDK patients, enhancing their management and care and potentially improving their overall health outcomes. 

## 5. Conclusions

In our study, we have observed that the frequency of peripheral Th1 cells was positively correlated with the CKD diagnosis period. There is strong evidence that, indeed, the immune system plays a significant role and may justify some of the disturbances and adverse events these patients are subjected to. Nevertheless, a better understating of the mechanisms of action leading to immune impairment and the proinflammatory environment is needed, even to clarify the relationship between immune responses and clinical outcomes in different renal pathologies.

## Figures and Tables

**Table 1:** Demographic and clinical characteristics of PD patients and controls.

	HD Patients n = 26	Controls n = 10	*p*-Value
Age (years)	**73.4 ± 12**	43 ± 11	<0.001
Male/Female [n (%)]	16/10 (61.5/38.5%)	6/4 (60/40%)	0.935
CKD primary cause [n (%)]			
Glomerulosclerosis/Diabetic nephropathy	8 (30.8%)	NA	
Renal hypoplasia (congenital) unspecified	2 (7.7%)	NA	
Pyelonephritis (interstitial nephritis)	3 (11.5%)	NA	
Renal vascular disease	4 (15.4%)	NA	
Other etiology	9 (34.6%)	NA	
CKD diagnosis (months)	75 ± 64.3	NA	
HD vintage (months)	56.3 ± 42.1	NA	
Kt/V	1.8 ± 0.4	NA	
Laboratory parameters			
Blood leukocyte counts (×10^3^ cells/μL)	6.5 ± 1.8	7.5 ± 3.2	0.348
Blood neutrophil counts (×10^3^ cells/μL)	**3.9 ± 1.4**	5.8 ± 0.9	<0.001
NLR	**2.0 ± 0.9**	3.4 ± 1.4	0.013
CRP (mg/L) *	**7.9 ± 12.4**	0.4 ± 0.3	0.012
Serum creatinine (mg/L)	**7.5 ± 2.2**	0.9 ± 0.1	<0.001
Pre-dialysis BUN (mg/dL)	111.8 ± 30.5	NA	
Post-dialysis BUN (mg/dL)	24.6 ± 10.6	NA	

Values are expressed as mean ± SD or number (n); *p*-values were determined by independent-sample *t*-test; statistically significant differences (*p* < 0.05) are identified in bold; HD, hemodialysis; CKD, chronic kidney disease; NA, not applicable; NLR, neutrophil–lymphocyte ratio; CRP, C-reactive protein; BUN, blood urea nitrogen. * These values were collected one month before the cytometric analysis of lymphocyte subpopulations; due to its relevance, we decided to include them in the analysis.

**Table 2:** Frequency and absolute values of total lymphocytes, T cells, CD4^+^- and CD8^+^-enriched T cells, and Th1 cells in PD patients and controls.

	HD Patients n = 26		Controls n = 10		*p*-Value	
	%	Cells/μL	%	Cells/μL	%	Cells/μL
total lymphocytes	25.7 ± 3.4	**1781.7 ± 756**	21.5 ± 5.7	1940 ± 670.3	0.011	0.570
T cells	73.7 ± 6.1	1231.7 ± 417.6	68.9 ± 7.6	1336.9 ± 482	0.055	0.530
CD4^+^	68.5 ± 4.5	844.9 ± 291.1	69.3 ± 6.9	917.5 ± 312.5	0.697	0.524
Th1	**14.2 ± 2.5**	**120.8 ± 47.3**	25.6 ± 7.6	235.7 ± 111.4	0.001	0.010
CD8^+^-enriched	31 ± 4.6	380.6 ± 144.1	30.7 ± 6.9	419.5 ± 210.7	0.891	0.383

Results are expressed as mean ± SD; *p*-values were determined by independent-sample *t*-test; statistically significant differences (*p* < 0.05) between HD patients vs. controls are identified in bold.

## Data Availability

To protect the patients’ privacy, no data supporting the obtained results will be made available.
